# TNA4OptFlux – a software tool for the analysis of strain optimization strategies

**DOI:** 10.1186/1756-0500-6-175

**Published:** 2013-05-03

**Authors:** José P Pinto, Rui Pereira, João Cardoso, Isabel Rocha, Miguel Rocha

**Affiliations:** 1 Department of Informatics/CCTC, University of Minho, Campus de Gualtar, Braga, 4710-057, Portugal; 2 IBB-Institute for Biotechnology and Bioengineering/Centre of Biological Engineering, University of Minho, Campus de Gualtar, Braga, 4710-057, Portugal; 3 SilicoLife, Lda., Avepark, Taipas, 4830, Portugal

**Keywords:** Strain optimization, Metabolic networks, Topological analysis, Open-source software, OptFlux

## Abstract

**Background:**

Rational approaches for Metabolic Engineering (ME) deal with the identification of modifications that improve the microbes’ production capabilities of target compounds. One of the major challenges created by strain optimization algorithms used in these ME problems is the interpretation of the changes that lead to a given overproduction. Often, a single gene knockout induces changes in the fluxes of several reactions, as compared with the wild-type, and it is therefore difficult to evaluate the physiological differences of the *in silico* mutant. This is aggravated by the fact that genome-scale models *per se* are difficult to visualize, given the high number of reactions and metabolites involved.

**Findings:**

We introduce a software tool, the Topological Network Analysis for OptFlux (TNA4OptFlux), a plug-in which adds to the open-source ME platform OptFlux the capability of creating and performing topological analysis over metabolic networks. One of the tool’s major advantages is the possibility of using these tools in the analysis and comparison of simulated phenotypes, namely those coming from the results of strain optimization algorithms. We illustrate the capabilities of the tool by using it to aid the interpretation of two *E. coli* strains designed in OptFlux for the overproduction of succinate and glycine.

**Conclusions:**

Besides adding new functionalities to the OptFlux software tool regarding topological analysis, TNA4OptFlux methods greatly facilitate the interpretation of non-intuitive ME strategies by automating the comparison between perturbed and non-perturbed metabolic networks. The plug-in is available on the web site http://www.optflux.org, together with extensive documentation.

## Findings

### Background

OptFlux [1] is an open-source software platform for Metabolic Engineering (ME) based on the use of stoichiometric models and constraint-based approaches to metabolic modeling. It has been developed with the aim to expand the user base of these methods beyond bioinformaticians and expert researchers, being characterized by its user-friendly interface. It supports several distinct methods for important tasks in ME, such as model handling and import/export, phenotype simulation of both wild-type and mutant strains, strain optimization, model visualization and pathway analysis.

To ensure that it could easily be adapted and extended to the user needs, both by its original and by third-party developers, OptFlux possesses a modular component-based architecture that allows an expansion of its functionalities through the use of plug-ins. This article presents a software tool, the Topological Network Analysis for OptFlux (TNA4OptFlux), a plug-in that adds to OptFlux the capability of creating and performing topological analysis over metabolic networks created from stoichiometric models. One of the major advantages of this tool is the possibility of using these tools in the analysis and comparison of simulated phenotypes, namely those coming from the results of strain optimization algorithms.

Indeed, one of the major challenges created by algorithms that search for non-intuitive ME solutions, such as gene knockouts, that improve the production of target compounds, is the interpretation of the changes that lead to overproduction. Often, one single gene knockout induces changes in the fluxes of dozens of reactions, as compared with the wild-type, and it is therefore difficult to evaluate the physiological differences of the *in silico* mutant. This is aggravated by the fact that genome-scale models *per se* are difficult to visualize, given the high number of reactions and metabolites involved.

Metabolic systems are composed by chains of reactions connected by shared metabolites. Consequently, these systems can be intuitively represented as networks. Indeed, the use of topological analysis over metabolic networks is an important approach to the analysis of metabolism, allowing the application of techniques from the field of graph theory to the study of the metabolic phenomena. The analysis of metabolism through the lenses of graph theory led to the discovery that metabolic networks share a similar architecture with other complex networks, indicating that similar laws may govern complex networks in nature and other fields. This, in turn, allows the outputs from the analysis of large and well-mapped non-biological systems to be used to characterize the intricate interwoven relationships that govern cellular functions. Within this context, metabolic networks have been characterized as being scale-free, small-world, modular and hierarchical networks [2].

A number of tools have been proposed for network analysis, spanning many tools that work with networks from distinct fields (e.g. Pajek [3]) and others more specifically devoted to biological network analysis. Within this last group, Cytoscape [4] has been emerging as the most popular, incorporating in its core a large set of features for network analysis and visualization and providing a development environment that allows its extension with specific plug-ins.

However, in spite of these existing tools, the field of network-based topological analysis has been traditionally separated from model-based phenotype simulation methods and Metabolic Engineering efforts. An exception is the area of pathway analysis, including the calculation and analysis of Elementary Flux Modes (EFMs) [5], but due to its complexity it can only be applied to small/medium scale networks.

Thus, the main purpose of TNA4OptFlux was to provide a bridge between these two families of methods. This feature distinguishes TNA4OptFlux from other previous work.

### Core functionalities of OptFlux

OptFlux provides an extensive set of tools for ME experts with user-friendly interfaces. It is able to create models by importing data in different formats including flat files (in an application specific format), CSV files, Systems Biology Markup Language (SBML) and other application specific formats (e.g. Metatool or Cell Designer). The models used in the platform are stoichiometric models and therefore include information on the set of reactions (their equations and reversibility), metabolites and, if available, gene-reaction associations.

OptFlux includes operations to run *in silico* phenotype simulations of the wild-type or mutant strains in different medium conditions, using several methods. These include the popular approach of Flux Balance Analysis (FBA) [6] and its variants (e.g. Parsimonious FBA (pFBA) [7]), as well as specific methods for the simulation of mutants. These algorithms calculate the values for the fluxes over the whole set of reactions in the model.

Users can also perform strain optimization, identifying sets of reaction deletions (or gene deletions if gene-reaction associations are available) that optimize a given set of objective functions related with desired industrial goals (e.g. the maximization of the production of a given compound). The algorithms used in this task, single and multiobjective Evolutionary Algorithms (EAs) or Simulated Annealing (SA), typically return several solutions, each consisting of a set of gene/reaction knockouts and the flux values for the reactions in the model, provided by phenotype simulation methods.

### Network representation

TNA4OptFlux supports three types of networks that can be generated from the same metabolic model. These three types can be easily created with minimal human input and the different configurations facilitate the analysis of network properties:

#### Reaction-compound networks

By default, a network is represented as a directed bipartite graph (a type of graph that has two kinds of vertices and where edges only connect vertices of different types). Here, reactions and metabolites are represented as vertices and the relationships of metabolite participation are given by directed edges, which either start (in case of production) or end (in case of consumption) in the reactions. This is the most complex network type, but this added complexity results in a graph that contains the maximal amount of information. Also, it does not have the difficulties associated with the other representations regarding the calculation of paths [8].

#### Compound-compound networks

In this case, a network is represented by a directed graph where vertices represent the metabolites and edges represent the reactions. Here, edges connect one metabolite to another if a reaction consumes the first metabolite and produces the second. This representation directly provides information about relationships between compounds, but it is not straightforward to use for path finding [8], because the paths found by search algorithms may have been obtained by passing through edges that represent the same reaction, thus resulting in mathematically correct but biologically unfeasible paths.

#### Reaction-reaction network

This representation is similar to the previous one, but with reactions represented by vertices and edges pointing from reactions producing metabolites to reactions using them as a substrate. This model is useful when the main point of focus is the study of the relationships between the reactions. However, it has similar problems for calculating paths as the former one.

**Figure 1:**
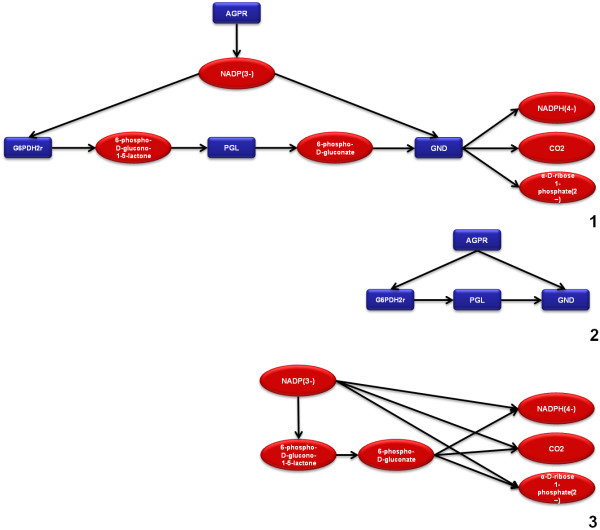
Illustration of the three types of network representation supported by TNA4OptFlux.

All the previous alternatives are based in directed graphs. Reversible reactions in all of the three representations use parallel directed edges pointing in opposite directions (Figure 1).

### Creating, visualizing and exporting networks

Networks in TNA4OptFlux are always created based on an existing metabolic model loaded in OptFlux and the user can select one of the three types of networks described in the previous section.

Regardless of the type, the metabolic networks obtained are typically quite complex graphs with a large number of vertices and edges. This is expected, since OptFlux typically works with genome-scale models that have a few thousand/hundreds of reactions and metabolites. Unfortunately, this places these metabolic networks well beyond the capability of even the most sophisticated current graph layout algorithms. For this reason, TNA4OptFlux does not implement a graphical visualization of the full network, such as it is common in network analysis tools. Instead, a set of tables is used to represent and characterize the network, including data on the network structure and the metadata associated with each vertex of edge.

**Figure 2:**
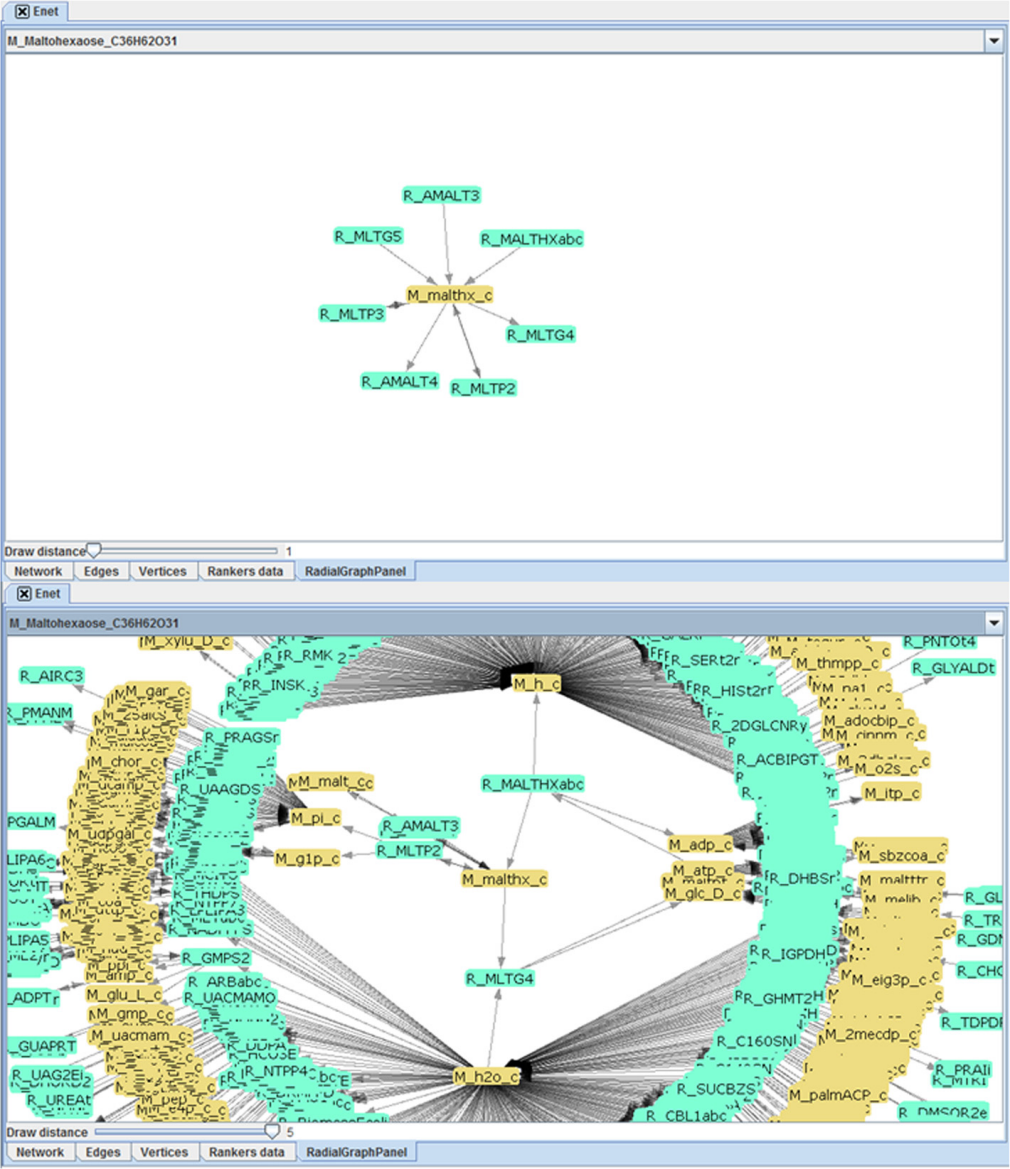
Illustration of the radial graph panel that allows to visualize metabolic networks centred in a metabolite.

While a visual representation of the full graph is not supported, TNA4OptFlux offers users some visual support, using the radial graph panel associated with a network. Here, it is possible to select a vertex and the plug-in draws a small graph with that vertex in the center and its neighbors in the radius. The network can then be navigated by clicking in one of the vertices in the radius, which redefines the central vertex and redraws the graph. Additionally, if for a given application visualizing only the immediate neighbors is insufficient, the distance of the vertices connected to the central vertex to be included in the graph (which is by default 1) can be adjusted up to 5, creating circles of growing radius. Illustrative examples are provided in Figure 2.

TNA4OptFlux is capable of exporting networks into several different kinds of file formats that are used by other network analysis applications. This allows users to complement the analysis conducted in this plug-in with other functionalities present in other software tools. Currently, the following main file formats are supported:

•XGMML format: An XML based network format, capable of storing all the metadata present in a network, facilitating the interchange between applications. The support for XGMML was included mostly for allowing the visualization of variation networks in other applications (see below), since unlike full metabolic networks, these are typically composed by only few independent modules. Cytoscape [4] is among the network analysis tools which support XGMML.

•Pajek [3] file format: Pajek is one of the most complete applications for network analysis, possessing a large number of methods of network analysis and manipulation.

### Topological analysis

Often, regardless of the system it represents, the topological analysis of a network is the first step in its study, revealing interesting properties of its structure. So, naturally, some topological analysis functionalities are provided by TNA4OptFlux. The topological analysis tools included are the degree analysis, the shortest path analysis and the calculation of distinct rankings based on centrality metrics.

#### Degree analysis

The degree analysis functionality provides the user a table view containing the degree of all vertices, discriminated by in- and out-degree. It can also be used to identify the list of neighbors of a selected vertex through a pop-up window, providing an idea of that vertex relative position in the network. A global analysis is also provided in the form of degree histograms (in table format). Finally, it calculates the degree distribution, a simple metric that nonetheless has proven in the past to be essential for identifying metabolic networks as scale-free [9].

#### Shortest path analysis

The determination of shortest paths can be used, for example, to give an idea, in a metabolic network, of the transformation an initial compound goes through until a desired substance is obtained. The plug-in supports the calculation of shortest paths between two selected vertices and can also be used to determine all the other vertices that a source vertex is connected to and the respective distances. Also, it includes a few global network metrics, such as the network diameter (longest shortest path in the network) and the mean shortest path calculated over all pairs of connected vertices in the network. This last metric can be useful to evaluate if the network can be called a small world network, i.e. if the mean length of the paths is smaller than what would be expected, a feature commonly associated with metabolic networks [10].

#### Ranking algorithms

This plug-in also includes a number of ranking algorithms, i.e. methods that provide a metric for each vertex, allowing to rank them by their importance. The most basic ranker is based on the degree of the vertices, but three more elaborate methods have been included: betweenness centrality (BC), closeness centrality (CC) and the hubs and authorities method (also known as Hyperlink-Induced Topic Search or HITS). The basic idea behind the BC is that an important vertex will lie on a high proportion of paths between other pairs of vertices in the network, while the CC considers that an important vertex is typically “close” to the other vertices in the network [11]. The HITS algorithm [12] calculates two different metrics for each vertex in a network: the hubness and the authority, being an algorithm originally developed to rate web pages based in their content.

### Locating active vertices

A reaction-compound network in TNA4OptFlux has a structure very similar to a Petri Net [13]. Taking inspiration from this similarity, a functionality called the location of active vertices was included in this tool. It uses an algorithm that starts with a set of “seed” metabolites (the active metabolites set) and from it determines the full set of reactions that can be active when this set of metabolites is present. This functionality works through an iterative process that, at each step, adds the metabolites that can be produced by the reactions in the model by using the current set of active metabolites as substrates. The reactions where all substrates are present in the active metabolites set are added to the active reaction set (which starts empty). The process continues until no further metabolites can be added to the active sets. The final result are sets of reactions which will be active and of metabolites which will exist in the system assuming an unlimited supply of the “seed” metabolites and sufficient time for the reactions to occur.

This functionality can help users determine if some metabolites are related with the production of other seemingly unrelated metabolites. A practical use is to define as active a set of external metabolites representing the components of the growth medium (nutrients) and determining all metabolites that can be potentially produced based in these initial seed metabolites.

### Filters

One of the difficulties of working with metabolic networks is the presence of the so-called ‘currency metabolites’ (or ubiquitous compounds) such as ATP, NAD and protons, which take part in a large number of reactions [14]. However, despite their frequency, currency metabolites often cannot be considered as valid intermediates for path finding, or for that matter, for establishing biologically meaningful network connections [8]. Thus, currency metabolites are often removed from metabolic networks before their analysis. However, since there is no commonly agreed definition of currency metabolites, often their removal is not as simple as it may seem. To address this problem and other similar tasks, filtering capabilities, which allow the removal of selected vertices based in user defined criteria, were included in TNA4OptFlux. The possible parameters for the removal of vertices include among others: a user defined list, degree values and minimum thresholds for the available ranking algorithms.

Since the degree is often the metric used to identify currency metabolites, many of the filtering operations are based in the degree, either by defining a degree threshold for vertex removal or by permitting the removal of the top **n** vertices with higher degree (where **n** is an user defined value). All these filtering operations can use either the value of degree or discriminate between out or in degree.

### Linking network analysis and phenotype simulations

Besides the analysis of metabolic networks associated with stoichiometric models, TNA4OptFlux has another more ambitious objective: to serve as a bridge between model-based and network-based analysis methods. Thus, a series of methods for the integration of simulation results with metabolic networks and processes for their subsequent analysis have been developed, being presented in this section.

#### Simulation filtering

In a typical phenotype simulation only a few of the metabolic reactions will take place, i.e. have a flux different from zero. This makes sense, since the bulk of the metabolism is composed by redundant or complementary metabolic capabilities whose occurrence depends on the conditions the organism is subject to and the available substrates.

To this effect, network filtering features were added, based on the results of phenotype simulation processes. These methods take as inputs a complete metabolic network (typically the original network created from the model) and the results of a phenotype simulation (using the same model), and output a sub-network containing only the parts of the metabolism that are active on that simulation. The obtained filtered network can be considered as a "snapshot" of the metabolism in the simulated conditions. The process is composed of two steps as follows:

1. The first step is to remove from the network all vertices corresponding to reactions with a predicted flux below a given threshold, considered to be inactive reactions.

2. After removing inactive reactions from the network, some vertices will be isolated from the rest of the network. This happens with metabolites that were only involved in reactions that were removed. These vertices are removed from the network.

These results of the simulation filtering can be seen as a "map", a sub-network containing all parts of the metabolism that are predicted to be used in a given condition. The study of the properties of this network can give insights into the behavior of the cell and the mechanisms involved in the metabolic responses in the conditions of the simulation.

#### Network comparison

Besides the analysis of a single network, the plug-in allows comparing the structure of different networks. This feature can be especially useful when analyzing several networks obtained from simulations over the same model. Some examples of fruitful application of this tool are the comparison of mutant strains with selected gene deletions versus wild-type strains, the comparison of simulations from distinct environmental conditions, etc.

Most of the network comparison functionalities are based in the provided network analysis metrics. These are used to identify the differences between the networks, by calculating all desired analysis metrics of those supported by the plug-in. To make a good use of computational resources, when two networks are compared, the plug-in checks which analysis metrics were applied to both and uses those in the comparison. Thus, to have a metric included in this comparison, the calculation needs to be previously executed for both networks.

The results of a network comparison are presented as a series of tables where the values of the metrics applied to individual vertices and for the networks as a whole can be observed. It should be noted that, when comparing networks, TNA4OptFlux assumes that vertices sharing the same id and type represent the same entity. This assumption is always true when the networks compared are derived from the same model. Care should be taken when comparing networks obtained from different models.

**Figure 3:**
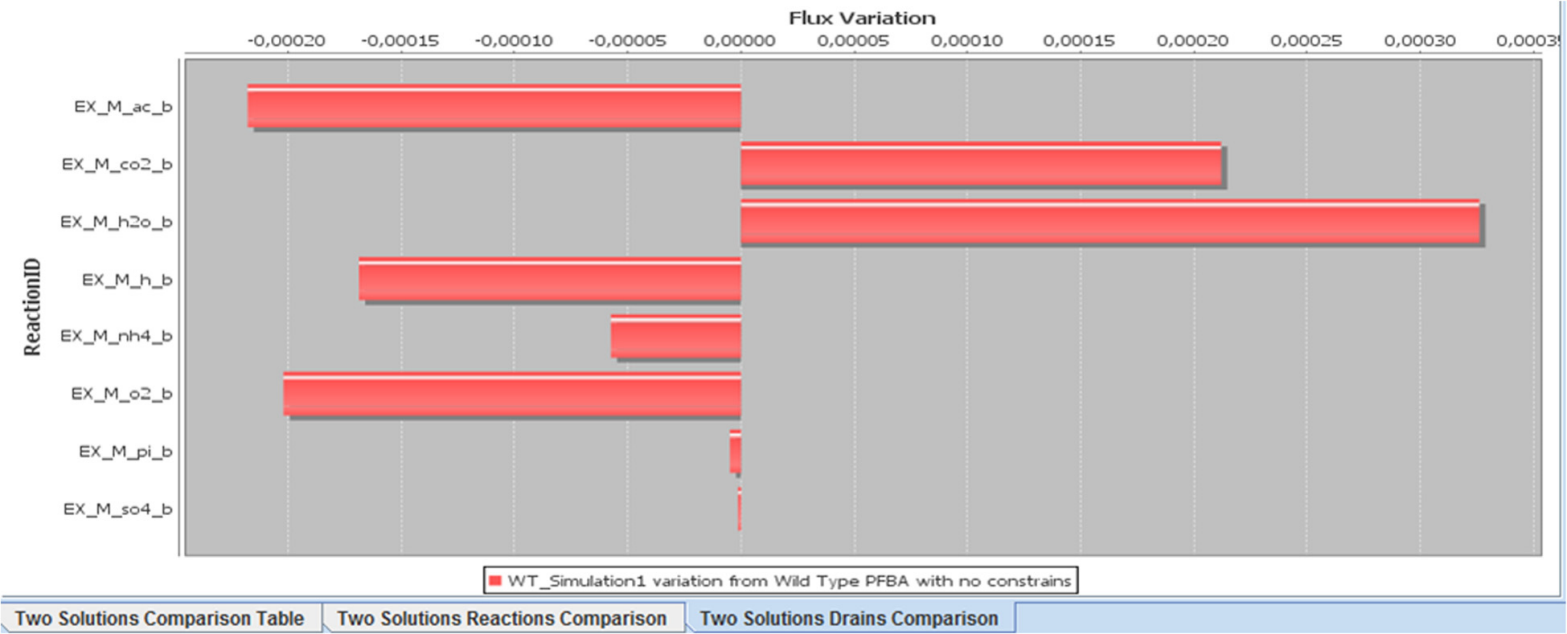
A bar plot of the drain reactions flux variations generated by TNA4OptFlux flux comparison feature for an example model.

When a phenotype simulation is performed for a model, flux values are kept in an object in the clipboard. In this plug-in, we have added a feature that, while not directly related to network analysis, can be of help in the comparison of different simulation results and complement the topological analysis tools. This feature allows a set of simulations to be selected and the results of the comparison among them to be presented both as a table and as bar plots that show the variation of flux values (see Figure 3).

#### Variation networks

A variation network is obtained by comparing a pair of networks obtained through simulation filtering, using the same base network and simulation results obtained under different conditions. The result is the creation of a third network containing the elements that differed between the compared networks (i.e. the variations).

The idea is to identify the components of a given metabolic system that change when the conditions inherent to the simulations change. Assuming the dimension of these variations to be significantly smaller than the networks themselves, variation networks should be easier to visualize and analyze than a full network.

The most important decision in this process is to select how the variations between the networks should be identified, i.e. what criteria should be used to decide on the components to be selected. Two methods are included in TNA4OptFlux:

1. *Exclusivity*: this is the simplest method but it has already proven useful in analyzing phenotype simulation results [15]. The criterion used is based on the identification of the exclusive reaction vertices in each network, i.e. those existing in one of the networks but not the other. After the identification of these reactions, the respective vertices are added to the variation network, together with the vertices corresponding to metabolites that they consume or produce, as well as the edges connecting them.

2. *Flux variation*: this method was developed since it was verified that methods based purely on network topology can be, in some cases, insufficient to capture more subtle metabolic flux variations. The flux variation is based in the comparison of flux values of the reactions present in both networks: if the absolute value of the difference in fluxes exceeds a user defined threshold (which can be an absolute flux value or a percentage) then the vertex corresponding to that reaction, as well as the ones corresponding to the metabolites which participate in it and the edges which connect them, are added to the variation network.

These two methods can be used independently or combined. After a variation network is created, it can then be analyzed as any other network using any of the tools available in the plug-in. There is, however, a point in which variation networks differ from other networks: they have metadata associated with their vertices identifying why each vertex was added to the network (values related to exclusivity, flux change, and flux values for each of the reactions). These tags are saved with the rest of the metadata when a variation network is exported into an XGMML file and can be used to visualize the type of variations associated with each vertex when using other network analysis tools. Indeed, some visualization applications can change the appearance (e.g. form, color) of the vertices based in these tags. For instance, in the case study described next, these tags were used to customize the color of the vertices when using Cytoscape [4] to visualize the variation network.

### Case studies

In order to evaluate the usefulness of the tools developed in this work on a practical example, we used a set of strain optimization results previously obtained and tried to analyse the mechanism behind a set of knockouts for the production of succinic acid (case study 1) and glycine (case study 2) using *E. coli* as a host. Our aim was to show that this plug-in can prove extremely valuable, especially when combined with network visualization software, such as Cytoscape.

#### Case study 1: succinic acid production with E. coli

Before beginning the analysis, a set of knockouts was selected by the authors from optimization results obtained using Optflux [16]. The set selected is reasonably complex, it is not too obvious nor consists of too many knockouts. In Table 1, a brief description is given of the set of knockouts elected as our case study 1. Apparently, there is nothing in common between the reactions in the table, and with the exception of succinate dehydrogenase, it is difficult to relate the inactivation of these reactions to succinate production.

**Table 1:** Set of knockouts and the corresponding chemical reactions used as case study

**Reaction**	**Stoichiometric equation**
Serine hydroxymethyltransferase	L-serine + tetrahydrofolate < = > glycine + 5,10-methylenetetrahydrofolate + H_2_O
pyridine nucleotide transhydrogenase	H^+^_e_ + NADH + NADP^+^ = > H^+^_c_ + NAD^+^ + NADPH
Succinate dehydrogenase	FAD^+^ + Succinate = > FADH_2_ + Fumarate
Transketolase I	D-erythrose-4-phosphate + D-xylulose-5-phosphate < = > D-fructose-6-phosphate + D-glyceraldehyde-3-phosphate

TNA4OptFlux allows to compare the flux distributions between the mutant and the wild-type simulations and extract relevant changes in the networks. Using the options available we can easily create a network and filter it accordingly to the flux values obtained for the wild-type or mutant simulations. Furthermore, we can also remove currency metabolites, such as H^+^ or ATP using the filtering functionality, in order to simplify the visualization of the network.

Among all the features available, the creation of variation networks is particularly important in the analysis of mutant phenotypes. This functionality allows the user to extract from the network only the reactions that change between two simulations (wild-type vs. mutant).

To analyse this case study, the first step was to simulate both the wild-type and selected mutant using pFBA [5], one of the methods available in OptFlux. Then, we filtered out the reactions with zero flux and removed some of the currency metabolites (H_2_O, H^+^, AMP, ADP and ATP) from the network, to simplify the graphical representation. Then, we computed the variation network between the wild-type simulation and the succinate producing mutant. The options were set to include the reactions exclusive to each simulation and the reactions where the flux had changed above 2 mmol/gCDW.h (~50% of the succinate production rate). The resulting variation network was exported to an XGMMLfile and within Cytoscape the visual parameters of the network were customized to help in the analysis. Reactions exclusive to the wild-type were painted in red, the ones exclusive to the mutant in blue, with increased flux in the mutant in green and with reduced flux in the mutant in orange.

**Figure 4:**
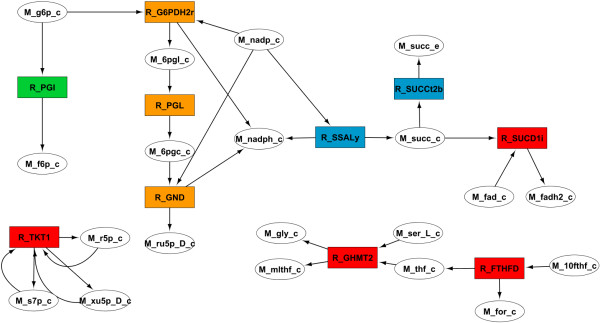
**Manually curated variation network between the wild-type and the succinate producing mutant described in Table**1**.** Wild-type exclusive reactions are shown in red, the ones exclusive to the mutant are in blue, while green and orange show reactions with increased or reduced flux in the mutant, respectively. **Abbreviations:** M_10fthf_c **– 10-Formyltetrahydrofolate,** M_6pgc_c**- 6-Phospho-D-gluconate,** M_6pgl_c**- 6-phospho-D-glucono-1,5-lactone**, M_f6p_c**- D-Fructose-6-phosphate,** M_fad_c**- Flavin adenine dinucleotide oxidized,** M_fadh2_c**- Flavin adenine dinucleotide reduced,** M_for_c**- Formate,** M_g6p_c**- D-Glucose-6-phosphate,** M_gly_c**- Glycine,** M_mlthf_c**- 5,10-Methylenetetrahydrofolate,** M_nadp_c**- Nicotinamide adenine dinucleotide phosphate,** M_nadph_c**- Nicotinamide adenine dinucleotide phosphate reduced,** M_r5p_c**- alpha –D-Ribose-5-phosphate,** M_ru5p_D_c**- D-Ribulose-5-phosphate,** M_s7p_c**- Sedoheptulose-7-phosphate,** M_ser_L_c**- L-Serine,** M_succ_c**- Succinate,** M_thf_c**- 5, 6, 7, 8-Tetrahydrofolate,** M_xu5p_D_c**- D-Xylulose-5-phosphate;** R_FTHFD**- formyltetrahydrofolate deformylase,** R_G6PDH2r**- glucose 6-phosphate-1-dehydrogenase,** R_GHMT2**- Serine hydroxymethyltransferase,** R_GND**- 6-phosphogluconate dehydrogenase,** R_PGI**- phosphoglucose isomerase,** R_PGL**- 6-phosphogluconolactonase,** R_SSALy**- succinate-semialdehyde dehydrogenase,** R_SUCCt2b**- succinate transporter,** R_SUCD1i**- succinate dehydrogenase,** R_TKT1**- transketolase.**

After generating the variation network, it was manually filtered to remove a few reactions known to have no significance to the solution based in the available knowledge about the system. The final result was the network shown in Figure 4.

This simplified view allowed us to infer that NADPH balance was the key factor behind succinate production. Firstly, the deletion of Transketolase I causes a decrease of flux through the pentose phosphate pathway, which means that much less NADPH will be produced here. Together with the inactivation of pyridine nucleotide transhydrogenase, it creates a shortage of this metabolite. In the figure, we can see that in order to compensate for this shortage, the model predicts the use of succinate-semialdehyde dehydrogenase, which produces succinate while reducing NADP^+^ to NADPH in the process. In order to promote the excretion of succinate outside of the cell, it is also vital to inactive succinate dehydrogenase.

**Figure 5:**
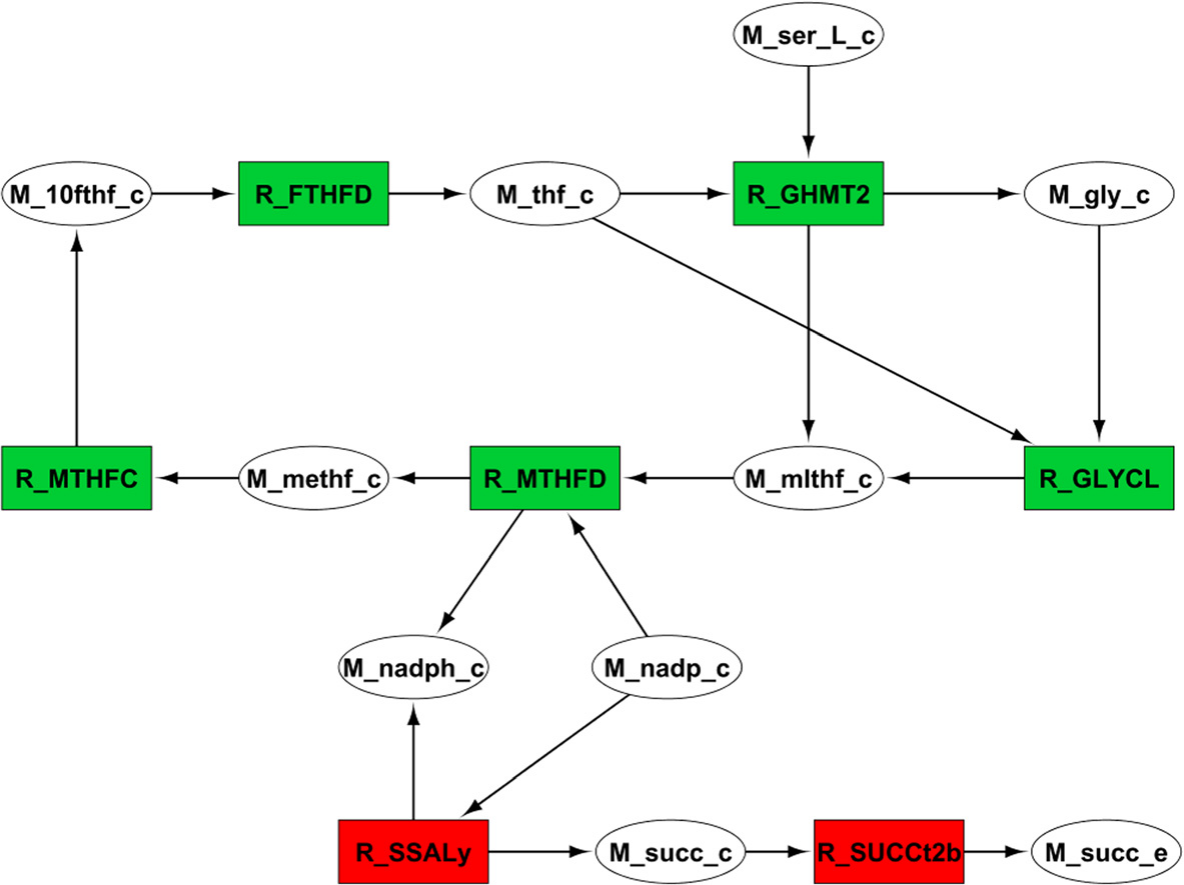
**Manually curated variation network between the succinate producing mutant and the triple mutant (R_TKT1, R_SUCD1i and R_THD2) described in Table**1**.** Wild-type exclusive reactions are shown in red, the ones exclusive to the mutant are in blue, while green and orange show reactions with increased or reduced flux in the mutant, respectively. **Abbreviations:** M_10fthf_c **– 10-Formyltetrahydrofolate,** M_gly_c**- Glycine,** M_mlthf_c**- 5,10-Methylenetetrahydrofolate,** M_methf_c**- 5,10-Methenyltetrahydrofolate,** M_nadp_c**- Nicotinamide adenine dinucleotide phosphate,** M_nadph_c**- Nicotinamide adenine dinucleotide phosphate reduced,** M_ser_L_c**- L-Serine,** M_succ_c**- Succinate,** M_thf_c**- 5, 6, 7, 8-Tetrahydrofolate;** R_FTHFD**- formyltetrahydrofolate deformylase,** R_GHMT2**- Serine hydroxymethyltransferase,** R_GLYCL**- glycine cleavage system,** R_MTHFC**- 5,10-methylene-tetrahydrofolate cyclohydrolase,** R_MTHFD**- 5,10-methylene-tetrahydrofolate dehydrogenase,** R_SSALy**- succinate-semialdehyde dehydrogenase,** R_SUCCt2b**- succinate transporter.**

The final deletion was not as easy to understand, because through the analysis of the variation network there was no apparent connection to the mechanism described above. Therefore, we tried to compare the distribution of fluxes between the succinate producing mutant and the triple mutant with an active Serine hydroxylmethyl-transferase reaction (R_GHMT2). We repeated the procedure followed before and obtained the network shown in Figure 5. The conclusion was that if only three of the deletions shown in Table 1 are implemented, a NADPH producing cycle is formed in the glycine production pathway in order to compensate for the shortage of this metabolite. This cycle is eliminated with the last knockout.

#### Case study 2: glycine production using E. coli

The workflow used for case study 2 was very similar to the one reported above. Firstly, a set of knockouts (Table 2) was chosen among the optimization results obtained by the authors using Optflux and the genome scale model iAF1260 [17]. Once again the objective was to select a set with a reasonable number of deletions, whose interpretation is not obvious.

**Table 2:** Set of knockouts and the corresponding chemical reactions used as case study 2

**Reaction**	**Stoichiometric equation**
Isocitrate lyase	Isocitrate = > Succinate + Glyoxylate
Glycine cleavage (complex)	Glycine + NAD^+^ + Tetrahydrofolate = > NH4^+^ + 5,10-Methylenetetrahydrofolate + NADH + CO_2_
Phosphoenolpyruvate carboxylase	Oxaloacetate + Phosphate < = > Phospho*enol*pyruvate + Bicarbonate
phosphoribosylglycinamide formyltransferase 2	5-phospho-ribosyl-glycineamide + Formate + ATP < = > 5'-phosphoribosyl-*N*-formylglycineamide + ADP + Phosphate + H^+^

At the first glance, only the deletion of the glycine cleavage systems appears to be related to glycine accumulation. Since the reaction consumes the target metabolite, it is logical that its deletion is important to promote glycine accumulation. However, the other deletions are quite diverse and extremely difficult to interpret without a careful analysis of the flux changes in the network.

**Figure 6:**
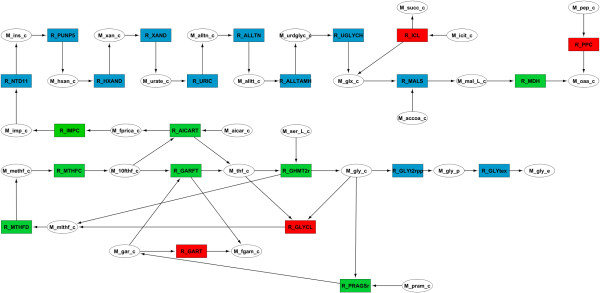
**Manually curated variation network between the wild-type and the glycine producing mutant described in Table**2**.** Wild-type exclusive reactions are shown in red, the ones exclusive to the mutant are in blue, while green and orange show reactions with increased or reduced flux in the mutant, respectively. **Abbreviations:** M_10fthf_c**- 10-Formyltetrahydrofolate,** M_accoa_c**- Acetyl-CoA,** M_aicar_c**- 5-Amino-1-(5-Phospho-D-ribosyl)imidazole-4-carboxamide,** M_alltn_c**- Allantoin,** M_alltt_c**- Allantoate,** M_fgam_c**- N2-Formyl-N1-(5-phospho-D-ribosyl)glycinamide,** M_fprica_c**- 5-Formamido-1-(5-phospho-D-ribosyl)imidazole-4-carboxamide,** M_gar_c**- N1-(5-Phospho-D-ribosyl)glycinamide,** M_glx_c- **Glyoxylate,** M_gly_c**- Glycine,** M_gly_e**- Extracellular Glycine,** M_gly_p**- Periplasmic Glycine,** M_hxan_c**- Hypoxanthine,** M_icit_c**- Isocitrate,** M_imp_c**- IMP, M_ins_c- Inosine,** M_mal_L_c**- L-Malate,** M_methf_c**- 5,10-Methenyltetrahydrofolate,** M_mlthf_c**- 5,10-Methylenetetrahydrofolate,** M_oaa_c**- Oxaloacetate,** M_pep_c**- Phosphoenolpyruvate,** M_pram_c**- 5-Phospho-beta-D-ribosylamine,** M_ser_L_c**- L-serine,** M_succ_c**- Succinate,** M_thf_c**- 5,6,7,8-Tetrahydrofolate,** M_urate_c**- Urate,** M_urdglyc_c**- (-)-Ureidoglycolate,** M_xan_c**- Xanthine,** R_AICART**- phosphoribosylaminoimidazolecarboxamide formyltransferase,** R_ALLTAMH**- allantoate amidohydrolase,** R_ALLTN**- allantoinase,** R_GARFT**- phosphoribosylglycinamide formyltransferase,** R_GART**- GAR transformylase-T,** R_GHMT2r**- glycine hydroxymethyltransferase**, R_GLYCL**- Glycine Cleavage complex**, R_GLYT2pp**- glycine transport in via proton symport (periplasm),** R_GLYtex**- Glycine transport via diffusion,** R_HXAND**- hypoxanthine dehydrogenase**, R_ICL**- Isocitrate lyase,** R_IMPC**- IMP cyclohydrolase,** R_MALS**- malate synthase,** R_MDH**- malate dehydrogenase,** R_MTHFC**- methenyltetrahydrofolate cyclohydrolase,** R_MTHFD**- methylenetetrahydrofolate dehydrogenase (NADP),** R_NTD11**- 5'-nucleotidase (IMP),** R_PPC**- phosphoenolpyruvate carboxylase,** R_PRAGSr**- phosphoribosylglycinamide synthase,** R_PUNP5**- purine-nucleoside phosphorylase (Inosine),** R_UGLYCH**- Ureidoglycolate hydrolase,** R_URIC**- uricase,** R_XAND**- xanthine dehydrogenase.**

TN4OptFlux was used to create filtered networks for each simulation (wild-type and mutant) as described for case study 1. Again, the reactions exclusive to either simulation and with values that changed above 2 mmol/gCDW.h (80% of the glycine production rate) were included in a variation network using the “create variation network” functionality. The resulting network was exported to Cytoscape and the nodes were colored as described for case study 1. Since two of the deleted reactions were not active on both simulations, they were added manually and colored red to help the analysis of what is their role in the flux redistribution. Finally, some nodes were removed by manual curation of the network according to the user’s know how and experience. The final result is shown in Figure 6 and was used to interpret the mechanism behind glycine accumulation in the mutant. From the figure, all the changes in the network can be analyzed in an integrated and user-friendly format, reducing the time of analysis when compared to the analysis of fluxes in a table format.

Using this variation network, it was possible to quickly conclude that two of the four deletions (Isocitrate lyase and Phosphoenolpyruvate carboxylase) are related to oxaloacetate metabolism, while the other two (Glycine cleavage complex and phosphoribosylglycinamide formyltransferase 2) are located in the glycine and one carbon metabolism. A careful analysis of Figure 6 showed that two anaplerotic reactions of oxaloacetate, Isocitrate lyase (R_ICL) and Phosphoenolpyruvate carboxylase (R_PPC), were inactivated and, therefore, another route for the regeneration of this essential metabolite needed to be activated. That sequence of reactions is clearly visible as a chain of blue nodes, originating from formamido-carboxamide (M_fprica_c) and culminating in the production of glyoxylate, which can be converted to L-malate by malate synthase (R_MALS) and then transformed into oxaloacetate. The reason for the redirection of M_fprica_c from one carbon metabolism to oxaloacetate was to increase the flux in phosphoribosylaminoimidazolecarboxamide formyltransferase (R_AICART), which is responsible for the synthesis of one of the precursors of glycine.

However, inactivating just R_ICL and R_PPC is not enough to drive the model to excrete glycine. If only these two reactions are deleted, some of the reactions in the glycine biosynthetic pathway and one carbon metabolism (lower part of Figure 6) could be used as a cycle of M_fprica_c production. Therefore, only when the Glycine cleavage complex (R_GLYCL) and phosphoribosylglycinamide formyltransferase 2 (R_GART) are deleted, is glycine produced as a by-product of M_fprica_c synthesis. Since either of these reactions can be used to recycle glycine, both deletions are required to promote glycine excretion.

To sum up, after deleting the anaplerotic reactions for oxaloacetate, another alternative is required to replenish the pool of this metabolite. In the genome scale model a chain of reactions originating from M_fprica_c can be used for this purpose. With two additional knock-outs (R_GART and R_GLYC), one in the glycine biosynthetic pathway and another in the one carbon metabolism, it is possible to couple glycine excretion to growth.

### Implementation details

OptFlux is implemented in the Java language, over AIBench [18], a software development framework originally from the University of Vigo that eases the implementation of scientific applications based on input-process-output workflows.

One of the major features of OptFlux is its modular architecture that allows the easy integration of novel components. This is normally achieved in the form of new plug-ins developed to be easily installed and interact with the core functionality of OptFlux. This created an environment where the TNA4OptFlux plug-in could be successfully developed, taking advantage on the implemented features, the development methodology (that follows the Model-View-Controller paradigm), the Graphical User Interfaces (GUI) and their components and the plug-in engine.

The development of this plug-in consisted in adding new *datatypes*, allowing the representation of the data objects from this work such as networks and analysis results, *operations* over those data (mainly analysis tools) and *views* (to allow visualizing networks and results).

The Java Universal Network/Graph Framework (JUNG) library [19] for graph creation and analysis was used as a basis for network operations within this plug-in.

In the structure provided by JUNG, a graph is represented in a way similar to its mathematical definition: a graph object, *g* containing two lists, one for the edges, *e*, and another for the vertices, *v*, together with the relationships among them. A useful feature of JUNG is that any kind of Java object can be used as an edge or a vertex. Taking advantage of this capability, objects that can include distinct metadata can be used as the elements of our networks. The JUNG library also includes implementations for many of the graph analysis tools required for biological network studies, easing the development of the application.

### Algorithms for shortest path calculation

In most of the methods for topological analysis implemented in the tool, the basis used were the algorithms available from the JUNG library. A specific algorithm was, however, needed for shortest path calculation. Indeed, the above described approach to represent reversible reactions causes some issues in the calculation of shortest paths between pairs of nodes. This problem consists in finding paths between two metabolites. The inclusion of two edges in opposite direction representing reversible reactions opens the possibility of including in a given path the reaction in which both metabolites participate as substrates or products. Naturally, these paths are meaningless from a biological point of view.

**Figure 7:**
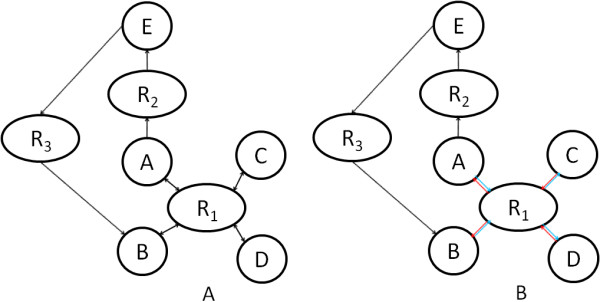
**Illustration of the SBBFS approach for shortest path calculation in metabolic networks (A) Using BFS, the shortest path between A and B is {A, R**_**1**_**, B}, a non interesting path, since A and B are in the same side of reaction R**_**1, **_**so they will always be consumed or produced together; (B) Using SBBFS, the shortest path between A and B will be {A, R**_**2**_**, E, R**_**3**_**, B}, a longer but biologically meaningful path.**

To address this issue a novel algorithm was developed, called set-based breadth-first search (SBBFS). This is a variation of the original breadth first search (BFS), by adding the notion of edge sets, i.e. edges have an associated attribute called the set that is kept in the edge metadata. The process of path finding must obey the condition that if a path enters a vertex through an edge of a set, it must exit that node through an edge belonging to the same set or an edge with no set. Figure 7 illustrates the approach followed providing an example.

With the SBBFS algorithm, if a metabolic network is correctly built, it is possible to identify all valid shortest paths from a selected vertex to all the vertices it is connected to. It should be noted that since SBBFS must store the paths and edge information, it is more memory expensive than the normal BFS.

## Conclusion

In this work, we propose to enrich the set of available tools for Metabolic Engineering by including in the open-source software platform OptFlux a new plug-in that makes the bridge between, on one hand, the methods based on stoichiometric models and constraints-based phenotype simulation approaches, and, on the other, topological analysis of metabolic networks. The main driving idea was to create tools that could help to understand and describe the metabolic strategies followed by the strains to achieve certain aims, both natural (e.g. production of biomass) or imposed (e.g. overproduction of a compound with industrial interest). Thus, the functionalities of the proposed plug-in were designed to make easier the tasks of analysing the results of phenotype simulation and strain optimization methods. The proposed case studies illustrated some tasks where the proposed plug-in aided in making life easier for the ME experts. TNA4 OptFlux proved to be a valuable tool to help uncover non-obvious mechanisms obtained from *in silico* simulations. When used together with visualization software such as Cytoscape, it reduced the time needed to understand how the redirection of fluxes leads to the accumulation of the target product.

## Availability and requirements

The OptFlux software is made available in the home page given below (current version is OptFlux 3). The TNA4OptFlux plug-in can be installed from within OptFlux through the interface for plug-in management. A wiki page is also available, providing diverse documentation both for OptFlux and for the plug-in.

More details:

Software name: Topological Network Analysis plug-in for OptFlux (TNA4OptFlux)

Project site: http://www.optflux.org

Direct link for plug-in documention: http://darwin.di.uminho.pt/optfluxwiki/index.php/OptFlux3:TNA

Operating system(s): Platform independent

Programming languages: Java

Other requirements: Java JRE 1.6.x, GLPK

License: GNU-GPL, version 3

## Abbreviations

BC: Betweenness centrality; BFS: Breadth first search; CC: Closeness centrality; CSV file: Comma-Separated Values file; EA: Evolutionary Algorithm; EFM: Elementary Flux Mode; FBA: Flux Balance Analysis; GUI: Graphical User Interface; HITS: Hyperlink-Induced Topic Search; JUNG: Java Universal Network/Graph Framework; ME: Metabolic Engineering; PFBA: Parsimonious Flux Balance Analysis; SBBFS: Set Based Breadth-First Search; SBML: Systems Biology Markup Language; SA: Simulated Annealing; TNA4OptFlux: Topological Network Analysis for OptFlux

## Competing interests

The authors declare that they have no competing interests.

## Authors’ contributions

JPP, IR and MR were involved in the conception of the software. JPP and MR were involved in the implementation. RP and IR idealized the case studies and analyzed the results. All authors helped to draft, reviewed and approved the final manuscript.
